# Initial radiographic tracheal ratio in predicting clinical outcomes in croup in children

**DOI:** 10.1038/s41598-019-54140-y

**Published:** 2019-11-29

**Authors:** Wen-Chieh Yang, Yu-Lung Hsu, Chun-Yu Chen, Yi-Chin Peng, Jun-Nong Chen, Yun-Ching Fu, Yu-Jun Chang, En-Pei Lee, Mao-Jen Lin, Han-Ping Wu

**Affiliations:** 1 Department of Pediatric Emergency Medicine, Children Hospital, China Medical University, Taichung, Taiwan; 2 Department of Medicine, School of Medicine, China Medical University, Taichung, Taiwan; 3 Devision of Pediatric Cardiology, Children’s Hospital, China Medical University, Taichung, Taiwan; 4 Laboratory of Epidemiology and Biostastics, Changhua Christian Hospital, Changhua, Taiwan; 5 College of Medicine, Chang Gung University, Taoyuan, Taiwan; 6 Division of Pediatric Critical Care Medicine, Department of Pediatrics, Chang Gung Memorial Hospital at Linko, Kweishan, Taoyuan, Taiwan; 7 Division of Cardiology, Department of Medicine, Taichung Tzu Chi Hospital, The Buddhist Tzu Chi Medical foundation, Taichung, Taiwan; 8 Department of Medicine, School of Medicine, Tzu Chi University, Hualien, Taiwan; 9 Department of Medical Research, Children’s Hospital, China Medical University, Taichung, Taiwan

**Keywords:** Radionuclide imaging, Paediatric research

## Abstract

Croup is the leading infectious disease resulting in pediatric upper airway obstruction. Our purpose is to analyze diverse features of neck radiographs could be seen as an objective tool to predict outcomes in patients with croup. One hundred and ninety-two patients were prospectively recruited in pediatric emergency department with diagnosis of croup. The initial Westley score (WS), presence of steeple sign, extent of narrowing, and narrowing ratio on soft tissue neck radiographs were determined before and after treatments. The extent of frontal narrowing, extent of lateral narrowing, frontal ratio (FR), and lateral ratio (LR) were investigated to predict clinical outcomes in patients with croup. The extent of frontal/lateral narrowing and LR had significant correlation with outpatient status. Almost 71% of patients with FR values below 0.23 stayed in the hospital longer, whereas nearly 98% of patients with FR vales above 0.65 could be discharged. About 85% of patients with LR below 0.45 hospitalized longer. The LR and FR were significantly correlated with the severity and admission rate in croup. The LR > 0.6 and FR > 0.65 may indicate low risk in patients with croup, whereas the FR < 0.23 or LR < 0.45 may indicate the need of stay in hospital for further treatment and monitor.

## Introduction

Croup, so-called acute laryngotracheobronchitis, mainly resulted in pediatric upper airway obstruction in the age of 6–48 months. Croup is caused by human parainfluenza virus types 1 and 2 (hPIV-1 and hPIV-2, individually) and partly by influenza with the characterize of inspiratory stridor, hoarseness, and barking cough. In the United States, hPIV-1 and 2 have also been associated with biennial surges in a great sum of children admitted to the emergency department with croup, which typically begin in autumn^1–3^.

In spite of the self-limiting clinical course, the acute onset of subglottic swelling in croup may adversely impact on sleep and feeding in affected young patients, while intubation may be necessary to prevent desaturation in severe cases. Several croup scores have been developed to objectively assess the severity of croup based on a variety of clinical factors. Among these, the Westley score (WS) is mostly accepted and has been generally utilized in numerous studies to estimate the therapeutic efficacy of croup treatment^2^. Theoretically, a good croup severity score would be certified with high inter-rater reliability, good construct validity, and good responsiveness to change. Nevertheless, it could be a big problem that significant deviations in WS exist amongst different examiners. Additionally, a physician may overvalue or underestimate the severity or may not accurately estimate croup in a bad-tempered, crying child. Although prior studies have reported a high kappa value for oxygen saturation, the usefulness of this value is limited, as decreased oxygen saturation is a rather late sign of croup^4–6^.

In the present study, we focused on radiographs of soft tissue in the neck, as subglottic tracheal narrowing (i.e., steeple signs) on anteroposterior images is a typical sign of croup. Furthermore, the neck x-ray is generally recommended to exclude other etiologies resulted in upper airway obstruction, for example foreign bodies, deep neck infection, or acute epiglottitis and is not routinely utilized for patients with croup^7,8^. Although such images may aid emergency physicians in determining the severity of upper airway obstruction and appropriate course of treatment, few studies have examined the utility of radiographic factors and WS score in the assessment of upper airway obstruction, and few have examined the ability of WS to predict the outcome^2,9^. No studies have aimed to reveal a correlation between findings on neck radiographs and outcomes of croup patients. Therefore, here, we were intended to estimate if diverse features of soft tissue neck radiographs could be seen as an objective outcome-predictor in croup patients.

## Methods

### Patients

We prospectively enrolled croup patients in the pediatric emergency department (PED) of a medical center between 2012 and 2014. Routine medical work-up was accomplished by the pediatric emergency team, which included six attending physicians and experienced nurses. Inclusion criteria were age under 18 years, diagnosis of croup, and willingness to undergo a soft tissue neck radiograph. The major medical decisions were made and medical data were recorded by one attending physician to prevent inconsistencies. The controversial cases would be discussed in the team to reach the final consensus. The study protocol was approved by the Institution Review Board and ethics committee of Changhua Christian Hospital, and it was conducted in accordance with the approved guidelines and the Declaration of Helsinki. Informed consent was obtained from a parent and/or legal guardian of children participating in the study. All methods were performed in accordance with the relevant protocols.

### Westley score

Variety of croup severity scores were created and used for physician to evaluate therapeutic efficacy^10^. Among them, WS is the most popular, validated and widely used tool since 1978. It quantified five individual factors: chest wall retraction, stridor, airway entry, cyanosis, and altered consciousness. The sum could be divided into four severity groups: mild, moderate, severe, and impending respiratory failure^11^. In this study, We measured the initial WS before treatment, 30–60 mins after each treatment and the time before discharge.

### Treatment

Following triage with vital signs obtained, included oxygen saturation with pulse oximetry, croup patients were evaluated and treated by the PED physician, who measured the initial WS, confirmed croup history, and performed a detailed physical examinations. All patients took the neck soft tissue x-ray before further management. WS was determined using such factors as stridor, decreased air entry, altered consciousness, cyanosis, and the degree of retraction. We treated each patient depending on their initial WS and prescribed inhaled epinephrine and 0.15 mg/kg dexamethasone (oral, intramuscular, or intravenous form). An O_2_ tent (cool mist) would be used if the SpO_2_ below to 92%. Rapid influenza tests would be done if the patients had high-grade fever and flu-like symptoms. Croup patients with mild severity took oral dexamethasone with or without inhaled epinephrine. Those with mild to moderately severity used inhaled epinephrine and dexamethasone (oral or intramuscular) and would be observed for almost 1 hour to monitor for any signs of improvement or deterioration. Croup patients with moderate to severe severity or whose obstruction signs persisted after initial treatment would stay in the pediatric observation unit (POU) or admitted for further treatment. In POU, one single dose of dexamethasone and inhaled epinephrine every 6 hours were administered until the WS was below 2. Patients would be discharged after their WS was <2.

Baseline clinical assessment, including age, sex, weight, SpO2, and WS were all recored. Outcomes were investigated in five patient groups: outpatient, return within 48 hours, POU, general ward admission, and ICU admission. The length of hospital stay (hours) were also recorded.

### Radiologic measurements

**Figure 1:**
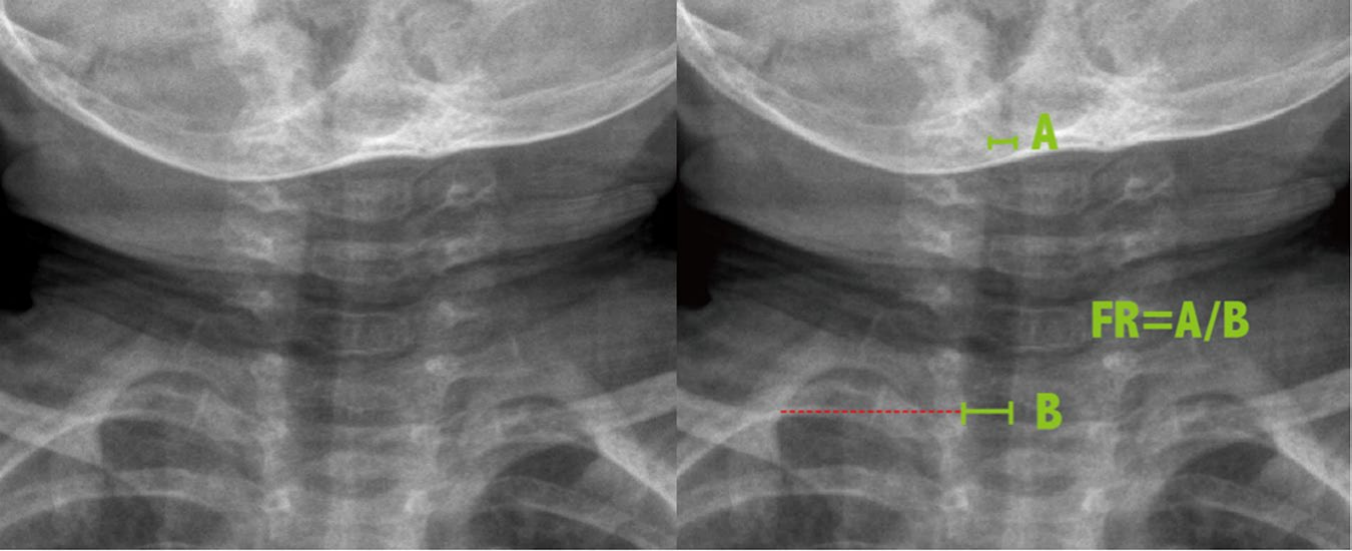
The measurement of frontal ratio.

**Figure 2:**
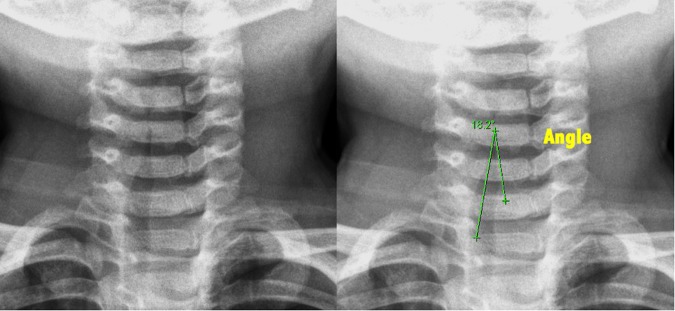
The measurement of steeple shaped angle.

**Figure 3:**
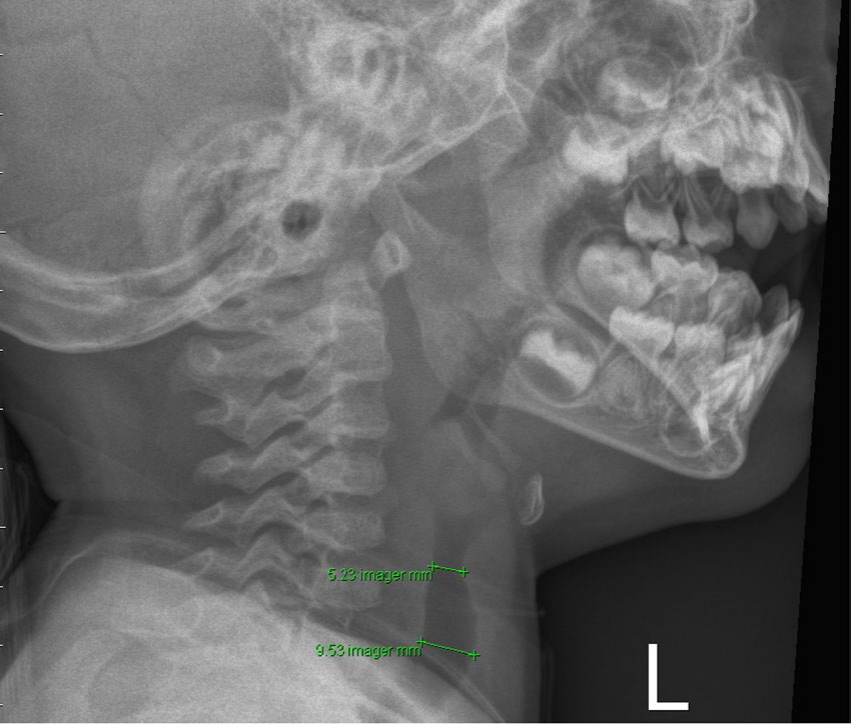
The measurement of lateral ratio.

We evaluated the presence of steeple signs, the extent of subglottic tracheal narrowing, and the narrowing ratio on the initial soft tissue neck radiographs of each patient. Frontal (FR) and lateral ratios (LR) were based on the initial soft tissue neck radiograph. The frontal ratio was measured from anteroposterior images. (Fig. 1) The ratio was defined as the narrowest tracheal width divided by the normal tracheal width. The normal tracheal width was recommended as 2 cm above the clavicle in an adult study^12^. But the level of 2 cm above the clavicle in some young children had reached the oral cavity, so we measured normal tracheal width at the following levels: at the level of clavicle, 1 cm above the clavicle, and 2 cm above the clavicle. The largest width was adopted as the normal tracheal width to determine the frontal ratio. For cases in which the narrowest width was 0 cm and steeple shaped angle could be measured, the angle was recorded as Fig. 2. The lateral ratio was measured from the lateral view of neck radiographs and defined as the narrowest tracheal width divided by the normal lateral tracheal width (Fig. 3).

### Statistical analysis

Fisher’s exact test or chi-square test as appropriate was used for comparing the correlation between outcomes and various clinical factors. Logistic regression analyses were used to evaluate the influence of various factors on patient outcomes. The correlation between WS and radiographic ratios was evaluated via correlation analysis. The cut-off points of the radiographic ratios were identified with receiver operating characteristic (ROC) analysis, which characterize with different cut-off values, including sensitivity, specificity, area under curve (AUC), positive likelihood ratio (LR^+^), and negative likelihood ratio (LR^-^). The AUC, calculated using the trapezoidal rule, was a standard measurement for the diagnostic value of the parameter. An optimal test result had a value of 1.0, while a valueless test result had a value of 0.5. The LR^+^ and LR^-^ were calculated for the best cut-off values. The criterion value indicated the value corresponding to the highest accuracy (minimal false negative and false positive results). The results of the descriptive analyses of independent variables were reported as percentages and mean ± S.D. A *p*-value less than 0.05 was considered statistically significant. Statistical analyses were performed using SPSS software (version 20.0; SPSS Inc., Chicago, IL, USA).

## Results

We initially included a total of 199 patients; however, three patients were subsequently excluded because of poor image quality, two owing to controversial croup diagnosis, and two because the criteria for admission were not based on disease severity. Eventually, 192 patients (age: 2.06 ± 1.76 years) with croup were enrolled in the study. and underwent neck radiographic imaging in our pediatric emergency department. The male to female was 2.5 to 1. Thirty-eight patients (19.8%) were ≤1 year old, while the remaining 144 patients (75%) were in the toddler to preschool age group. All patients recovered from croup without any sequelae. The mean WS of all patients was 3.2 ± 1.3. One hundred and thirty-seven patients (71.4%) had WS of 1–3 and 55 patients (18.2%) had WS above 3 (Table 1). Croup was associated with fever in 80.2% of patients (N = 154). Thirty-four patients (17.7%) presenting with croup had a past history of croup. Steeple signs were observed on the radiographic images of thirteen patients (6.8%), and the mean tracheal angle measured 33 ± 9 degrees. The mean frontal and lateral ratios were 0.28 ± 0.15 and 0.60 ± 0.17, respectively. A total of 138 patients (71.9%) received outpatient care after initial treatment in the PED, while 43 patients remained in the POU for further treatment and observation within 24 hours, 19 patients hospitalized to our general ward, and two patients were admitted to our ICU for intensive care. Among the two patients hospitalized to the ICU, one had been intubated in our PED, while the other had been intubated in a separate local hospital. The latter patient was extubated during ambulance transport for unknown reasons and was not re-intubated in our hospital. The mean length of stay for all patients was 54.4 ± 57.8 hours (N = 54), 14.8 ± 7.8 hours in the POU group, 115.0 ± 34.5 hours in the general ward group, and 60.0 ± 50.9 hours in the ICU group.

**Table 1:** Patient characteristics.

	Croup patients
	N (%/Mean ± SD)
Age (years)	192 (2.06 ± 1.76)
<1	38 (19.8)
1–6	144 (75.0)
>6	10 (5.2)
Female/Male	54/138 (1:2.5)
Initial Westley score	192 (3.2 ± 1.3)
1–3	137 (71.4)
4–6	54 (17.7)
≥7	1 (0.5)
Fever	154 (80.2)
Croup hx (+)	34 (17.7)
Steeple sign	13 (6.8)
Tracheal Angle*	33.07 ± 8.79
FW (cm)	2.03 ± 1.10
FR	0.28 ± 0.15
LW (cm)	3.79 ± 1.43
LR	0.60 ± 0.17
Outpatient	138 (71.9)
LOS-total (hours)	54 (54.4 ± 57.8)
LOS-POU (hours)	43 (14.8 ± 7.8)
LOS-ward (hours)	19 (115.0 ± 34.5)
LOS-ICU (hours)	2 (60.0 ± 50.9)

*Tracheal angle of steeple sign.

FW: frontal narrowest width; LW: lateral narrowest width.

FR: Frontal Ratio, LR: Lateral Ratio.

LOS: length of hospital-stay.

The comparison of radiographic factors between the outpatient group and the hospitalization group is presented in Table 2. No significant correlation was observed between age and rate of admission in any group (*p* value = 0.185). Similarly, no significant correlation was observed between the presence of steeple signs and severity/admission rate (*p* value = 0.523). Among other radiographic factors, the frontal narrowing width (*p* value = 0.002), the lateral narrowing width (*p* value = 0.003), LR (*p* value = 0.015), LR above 0.6 (*p* value = 0.009), and the product of FR and LR (*p* value = 0.041) were significantly correlated with outpatient status. We did not include return within 48 hours as a factor, as only two patients returned within 48 hours, one of whom remained in the POU for further observation.

**Table 2:** Correlation between various factors and patient outcome.

Variables	Outpatient		*P* value
	No (n = 54)	Yes (n = 138)	
Age < 1	15 (27.8)	23 (16.7)	0.185
Age 1–6	36 (66.7)	108 (78.3)	
Age > 6	3 (5.6)	7 (5.1)	
Age(years)	1.84 ± 1.86	2.14 ± 1.71	0.275
Steeple sign	5 (9.3)	8 (5.8)	0.523
FW (cm)^Ψ^	1.67 ± 0.89	2.17 ± 1.14	0.002
FR	0.25 ± 0.16	3.98 ± 1.43	0.058
LW (cm)^Ψ^	3.30 ± 1.32	3.98 ± 1.43	0.003
LR^Ψ^	0.55 ± 0.19	0.62 ± 0.16	0.015
LR > 0.6^Ψ^	36 (66.7)	63 (45.7)	0.009
FR*LR (or LR*LR, if FR = 0)^Ψ^	0.16 ± 0.13	0.20 ± 0.12	0.041
Fever	47 (87.0)	107 (77.5)	0.137
Croup hx (+)^Ψ^	4 (7.4)	30 (21.7)	0.019

P-value by Chi-square test or Fisher’s exact test when appropriate.

P-value of mean ± SD by Student’s t-test.

FW: frontal narrowest width; LW: lateral narrowest width.

FR: Frontal Ratio, LR: Lateral Ratio.

LOS: length of hospital-stay.

Ψ: p-value < 0.05.

The results of the correlation analysis between radiographic ratios and the length of stay are presented in Table 3. Radiographic factors were modestly correlated with the length of stay (LOS).

**Table 3:** Correlation of clinical and radiographic severity factors.

	(n = 192)	FW	FR	LW	LR	LOS (hrs)
FW	r	1.000	0.891	0.515	0.336	−0.218
	p-value		<0.001	<0.001	<0.001	0.002
FR	r	0.891	1.000	0.329	0.343	−0.184
	p-value	<0.001		<0.001	<0.001	0.011
LW	r	0.515	0.329	1.000	0.644	−0.252
	p-value	<0.001	<0.001		<0.001	<0.001
LR	r	0.336	0.343	0.644	1.000	−0.189
	p-value	<0.001	<0.001	<0.001		0.009
LOS (hrs)	r	−0.218	−0.184	−0.252	−0.189	1.000
	p-value	0.002	0.011	<0.001	0.009	

r: Spearman’s rho correlation coefficient.

FW: frontal narrowest width; LW: lateral narrowest width.

FR: Frontal Ratio, LR: Lateral Ratio.

LOS: length of hospital-stay.

Logistic regression analysis was performed to evaluate the impact of various factors on outpatient status (Table 4). Patients within the first age had higher outpatient tendency than toddler and preschool age group (1 to 5.9 years old), and those with higher LR and FR values were discharged to outpatient care only after initial treatment in the PED. Among all significant factors, the highest odds ratios were observed for FR (odds ratios: 15.50) and LR (odds ratios: 10.19).

**Table 4:** Logistic regression analysis of factors associated with outpatient status.

	Outpatient	Univariate analysis			Multiple analysis (adjusted)		
	N(%) / Mean ± SD	OR	95% C.I.	*P* value	OR	95% C.I.	*P* value
**Age**							
<1 y/o	23 (60.5)	1.00		0.22	1.00		
1–6 y/o^Ψ^	108 (75.0)	1.96	0.92–4.15	0.08	2.79	1.10–7.09	0.03
>6 y/o	7 (70.0)	1.52	0.34–6.83	0.58	0.70	0.10–4.67	0.71
FR^Ψ^	0.29 ± 0.14	8.87	0.91–86.42	0.06	15.50	1.09–221.35	0.04
LR^Ψ^	0.62 ± 0.16	10.19	1.53–67.95	0.02			

OR: Odds ratio.

FR: Frontal Ratio, LR: Lateral Ratio.

Ψ: p-value < 0.05.

ROC curve analysis was used to identify the cut-off points of radiographic ratios in the prediction of outpatient status/hospitalization (Table 5). Among patients with FR values less than 0.23, approximately 71% hospitalized for further care (sensitivity: 52%; specificity: 71%; AUC: 0.61). Among patients with FR higher than 0.65, approximately 98% were discharged to outpatient treatment. Among patients with LR values less 0.45, approximately 85% patients hospitalized for further care (sensitivity: 37%; specificity: 85%; AUC: 0.61). Among patients with LR values higher than 0.6, approximately 96% were discharged to outpatient treatment.

**Table 5:** Receiver operating characteristic (ROC) curve analysis of radiographic factors for the prediction of outpatient status.

Criterion values and coordinates of ROC curve						Area under the ROC curve			
Variable	Value	Sens	Spec	LR^+^	LR^−^	AUC	SE	95% C.I.	*P* value
FR^Ψ^	0.23	0.52	0.71	1.79	0.68	0.61	0.05	0.52–0.70	0.02
	0.03	0.09	0.94	1.60	0.96				
	0.65	0.98	0.01	1.00	1.36				
LR^Ψ^	0.45	0.37	0.85	2.43	0.74	0.61	0.05	0.52–0.70	0.018
	0.03	0.04	0.92	0.45	1.05				
	0.6	0.96	0.04	1.00	1.02				

Ψ: p-value < 0.05.

FR: Frontal Ratio, LR: Lateral Ratio; LR: likelihood ratio.

## Discussion

By the analysis of soft tissue radiographs, we observed that radiographic factors associated with tracheal width were significantly correlated with clinical severity and outcomes in patients with croup treated in a PED.

The point at which tracheal width is measured is a critical factor in determining the usefulness of radiographic images for the evaluation of croup severity. Dr. Breatnach suggested that normal tracheal width in the anteroposterior view should be measured approximately 2 cm above the clavicle^12^. However, the level 2 cm above the clavicle in young children were usually above the narrowed area and even reached the oral cavity. Therefore, our findings indicate that the most appropriate area for measuring normal tracheal width is at the level of the upper clavicular border, after comparing measured tracheal width at three locations.

The LR and FR ranges for patients of the present study were 0.24–0.98 and 0.00–0.81, respectively. Although patients with lower LR and FR values had higher rates of admission and WS scores, the steeple sign on anteroposterior images was not significantly correlated with croup severity or patient outcome. Among the thirteen (6.7%) patients who presented with steeple signs, only five (38%) were admitted. Although further studies involving larger patient populations are required for meaningful statistical analyses, we also observed larger LR values for patients with mild croup exhibiting steeple signs. This phenomenon may have resulted from frontal dislocation of the swollen subglottic tissues, in turn resulting in a transformation error on two-dimensional radiographs. Accordingly, steeple sign should not be used to indicate severity of croup patients.

It is difficult to obtain perfect anteroposterior images in young patients, as even slight head-tilting may influence tracheal width in the anteroposterior view, although the effect is less severe on lateral than frontal images. However, our findings indicate that LR and FR values can be used to determine whether most patients with croup can be discharged after initial treatment (OR: 10.19 / 15.50). Larger LR & FR values were statistically correlated with outpatient care. Furthermore, ROC curve analysis suggested that patients could be grouped into three levels of severity based on radiographic findings: mild, LR >0.6 or FR >0.65; moderate, LR between 0.45–0.6 or FR between 0.23–0.65; and severe, LR <0.45 or FR <0.23. Thus, patients in the mild group can be discharged following single doses of inhaled epinephrine and dexamethasone. Indeed, our findings indicate that patients could be discharged when initial LR values were above 0.6 (sensitivity: 96%; specificity; 1%, *p* = 0.018) and FR values were above 0.65 (sensitivity: 98%; specificity; 4%, p = 0.02). However, patients with LR values below 0.45 (sensitivity: 85%; specificity: 37%) or FR values below 0.23 (sensitivity: 71%; specificity: 52%) should be admitted following initial treatment with single doses of inhaled epinephrine and dexamethasone.

Otherwise, the relationship between the radiographic findings and other clinical findings were modestly correlated. We also observed no significant correlations between radiographic factors and age. In a previous study, the highest rate of admission from the ED was observed in children at least 1 year of age, followed by children younger than 1 year^3^. However, Fitzgerald and Kilham considered age <6 months to be one of several predisposing factors for admission following initial treatment for croup^13^. In the present study, we observed no significant correlation between age and rates of admission or croup severity in patients under 1 year of age. Furthermore, no patients under 1 year of age returned within 48 hours due to worsening of clinical symptoms. Patients in the toddler to preschool age group (1–6 years old) had a higher chance of outpatient status (OR: 2.79).

Several studies have aimed to determine the point at which relief from stridor symptoms occurs following initial treatment with inhaled epinephrine and oral/intramuscular/intravenous dexamethasone. Most researchers have agreed that patients typically require 2–3 days to recover, although up to 1 week may be necessary to relive obstruction of the upper airway in some patients^13–16^. In the hospitalization group of the present study, approximately 15 hours was required for patients with WS above 4 to achieve a WS of 2 following admission to the POU. Although recovery rate was not analyzed in the present study, the length of stay ranged from 1 to 37 hours, and the major predicting factors were initial WS and radiographic factors. No patients in the POU group experienced a clinically biphasic course, and earlier symptom relief was noted in patients who had received earlier treatment with medication. Previous studies have indicated that the effects of steroid treatment should be evident 4 to 6 hours following treatment in patients with mild to moderately severe croup^7^. In contrast, previous reports have indicated that the effects of oral dexamethasone at 0.15 mg/kg become evident within 30 minutes^15^. In our study, no patients in the outpatient group experienced a worsening of obstruction symptoms 30 minutes after initial treatment. Indeed, only two patients (1.5%) returned within 48 hours.

The present study possesses some limitations of note. Most importantly, we did not obtain follow-up radiographic images after treatment to ensure that children received minimal exposure to radiation. A larger sample size of study may be necessary in the further research to produce adequately powered analyses in older patients.

## Conclusion

Our findings indicated that radiographic factors may objectively assess the severity of croup. The LR and FR were significantly correlated with the severity and admission rate in croup. The LR > 0.6 and FR > 0.65 may indicate low risk in patients with croup, whereas the FR < 0.23 or LR < 0.45 may indicate the need of stay in hospital for further treatment and monitor^17^.

## Data Availability

The data that support the findings of this study are available, on reasonable request, from the corresponding author.
